# Multi-heuristic dynamic task allocation using genetic algorithms in a heterogeneous distributed system

**DOI:** 10.1016/j.jpdc.2010.03.011

**Published:** 2010-07

**Authors:** Andrew J. Page, Thomas M. Keane, Thomas J. Naughton

**Affiliations:** a Wellcome Trust Sanger Institute, Wellcome Trust Genome Campus, Cambridge, CB10 1SA, UK; b Department of Computer Science, National University of Ireland, Maynooth, Co.Kildare, Ireland; c University of Oulu, RFMedia Laboratory, Oulu Southern Institute, Vierimaantie 5, 84100 Ylivieska, Finland

**Keywords:** Scheduling, Genetic algorithms, Heterogeneous, Distributed computing

## Abstract

We present a multi-heuristic evolutionary task allocation algorithm to dynamically map tasks to processors in a heterogeneous distributed system. It utilizes a genetic algorithm, combined with eight common heuristics, in an effort to minimize the total execution time. It operates on batches of unmapped tasks and can preemptively remap tasks to processors. The algorithm has been implemented on a Java distributed system and evaluated with a set of six problems from the areas of bioinformatics, biomedical engineering, computer science and cryptography. Experiments using up to 150 heterogeneous processors show that the algorithm achieves better efficiency than other state-of-the-art heuristic algorithms.

## 1 Introduction

Many heuristic algorithms exist for the task allocation problem, but most are limited to specific cases [10]. The use of evolutionary algorithms in scheduling, that apply evolutionary strategies from nature, allows for the fast exploration of the search space of possible schedules. This allows for good solutions to be found quickly and for the scheduler to be applied to more general problems. The genetic algorithm (GA) [6] evolutionary strategy has been shown to consistently generate more efficient solutions than other evolutionary strategies when applied to scheduling in heterogeneous distributed systems [2].

Many researchers have investigated the use of GAs to schedule tasks in homogeneous [7,15,16,30] and heterogeneous [1,2,18,27,29] multi-processor systems with some success. However, the generality of these solutions are often reduced because of the assumptions made; (i) calculating schedules off-line in advance [1,2,7,27,29], (ii) a priori knowledge of communication times and task processing times [1,2,7,27,29], (iii) instantaneous message passing [30], (iv) all processors are homogeneous [7,30], and are dedicated to the distributed system [1,7,10,25,27–31]. All of these assumptions limit the applicability of a scheduler in a real-world distributed system. It is our belief that if a scheduler is to be made applicable to real-world distributed computing environments and problems, then it should not make any prior assumptions about resource homogeneity or availability.

In this paper a scheduling strategy is presented that uses a GA to schedule a set of heterogeneous tasks on to a set of heterogeneous processors in an effort to minimize the total execution time. It operates dynamically, allowing for tasks to arrive for processing continuously, and considers variable system resources, which has not been considered by other dynamic GA schedulers. To allow for efficient schedules to be produced quickly, the scheduler utilizes 8 heuristics, reducing the probability of processors becoming idle while waiting for a schedule to be generated. The scheduler has been implemented on a real-world distributed system and tested on 150 non-dedicated heterogeneous processors, with a variety of real-world problems from bioinformatics, biomedical engineering, computer science and cryptography. This paper significantly extends [24], which presented a GA scheduling algorithm, enhanced by a single heuristic. Simulated experiments showed that this method could be used to create efficient schedules, however this scheduler had all system and task information available to it in advance, and the processing resources, communication resources and the task computation requirements were drawn from standard distributions. The major contributions of this paper include: on-line estimation of resources, dealing with varying resources, dynamically modeling task execution time distributions, and providing an efficient method for scheduling in real-world heterogeneous distributed systems with zero advanced knowledge.

## 2 Genetic algorithm

We have created an algorithm which can adapt to varying resource environments utilizing a multi-heuristic GA (see Algorithm 1), originally based on the homogeneous dynamic load-balancing algorithm in [30] and an extension of [24]. We wish to schedule an unknown number of tasks for processing on a distributed system with a minimal total execution time, otherwise known as makespan.

**Figure:**
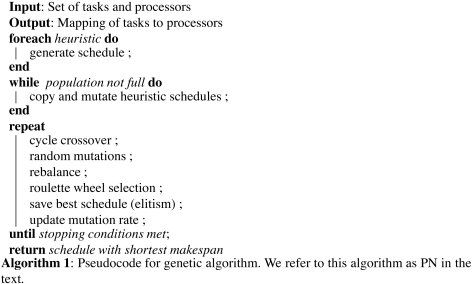

The set of processors of the distributed system is heterogeneous. The available network resources between processors in the distributed system can vary over time. The availability of each processor can vary over time (processors are non-dedicated). Tasks are indivisible, independent of all other tasks, arrive randomly, and can be processed by any processor in the distributed system.

When tasks arrive they are placed in a queue of unscheduled tasks. Batches of tasks from this queue are scheduled on processors during each invocation of the scheduler. The queue of unscheduled tasks can contain a large number of tasks. If all of these tasks where to be scheduled at once, the scheduler could take a long time to find an efficient schedule. To reduce the execution time of the scheduler and reduce the chance of processors becoming idle, we only consider a subset of the unscheduled tasks, which we call a batch. A larger batch will usually result in a more efficient schedule [30], but will incur a longer running time. To do this we dynamically set the batch size according to the estimated amount of time until the first processor becomes idle (further details can be found in [24]).

Each idle processor in the system requests a task to process from the scheduler, which it processes and returns. The scheduler contains a queue of future tasks for each processor, and when a request for work is received the task at the head of the corresponding queue is sent for processing. A processor does not contain a queue of tasks; because network resources are limited and processing resources are not dedicated. We also wish to avoid repeatedly issuing the same task multiple times, e.g., when a machine is switched off. When the server has spare resources it continues to improve the planned schedule in a non-preemptive fashion (running tasks are not moved). This then allows for a more efficient operation of the system when a task exceeds its estimated running time. The server stores information about the processors, tasks and communication channels. This information is then used to estimate the properties of the system and the resource requirements of the tasks to be processed. This is presented in more detail in [22].

### 2.1 Encoding

**Fig. 1:**
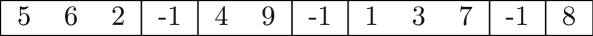
Encoding of a schedule within the GA, with −1 delimiting processor queues. Each number corresponds to a unique task ID, thus allowing for a mapping of tasks to processors.

Each schedule is encoded as a string of characters, using the same analogy as the encoding of DNA in nature. A single solution is referred to as a chromosome, and a set of multiple possible solutions is referred to as a population. Fig. 1 shows the encoding used within the GA. Each number represents a unique task identifier, with −1 being used to delimit different processor queues. This encoding allows for the execution order of tasks to be defined on each processor, which allows for precedence constraints (not covered in this research). If the execution order of tasks was not required to be defined, a simpler encoding can be used, where the index of each character corresponds to a task, and the character itself corresponds to a processor.

### 2.2 Fitness function

A fitness function attaches a value to each chromosome in the population, which indicates the quality of the schedule. It comes from the evolutionary principle of ‘survival of the fittest’, where the organisms with the best characteristics for their environment have a better chance of surviving to the next generation than weaker organisms, which are less adapted to their environment. We use a localized makespan to delineate fitness. Simply taking the makespan of a solution only considers the total execution time, however a well balanced load distribution is also a desirable property, which will also lead to a lower makespan. Thus we have developed a fitness function which utilizes both. The localized makespan looks at when each processor will become idle next, and adds on the time to process each task in the proposed schedule. The processors with the largest and smallest processing times are then identified. If these times are the same, it indicates a perfectly balanced schedule. As the difference becomes greater, so does the load imbalance, which also effects the efficiency of the resource utilization. The localized makespan of the yth batch of tasks is $L_{x}=\max_{j=1}^{m}\left(\sum_{i=1}^{n_{y}}A_{i}^{j}+B_{i}^{j}\right)-\min_{j=1}^{m}\left(\sum_{i=1}^{n_{y}}A_{i}^{j}+B_{i}^{j}\right)$ where $n_{y}$ is all of the tasks, up to and including the yth batch of tasks, A is the processing time of a task, B is the communication overhead of the task, and x is a schedule from the population. The fitness value of chromosome x is

(1) $$\left[F_{x}=\left\{ \begin{array}{ccc} 1 & \text{:} & L_{x}=0\\ 1/L_{x} & \text{:} & \text{otherwise ,} \end{array}\right.\right]$$

and $F_{x}=\left[0,1\right]$. A larger value indicates a better or fitter schedule.

### 2.3 Multiple heuristics

We use eight simple heuristics to create an initial population within the GA scheduler. We chose to use 4 heuristics, along with 4 variations, which are very simple and commonly found in real-world systems, often with only slight variations and/or different names. Two are batch heuristics and 2 are immediate mode heuristics. We hypothesise that using more heuristics will improve the overall initial population, however this requires further research and is beyond the scope of this paper. The remainder of the population is generated using random permutations of these heuristics. The use of multiple heuristics in our initial population provides the GA with reasonable starting solutions, compared to starting with a completely randomly generated initial population. By employing elitism, the GA will always produce a solution which is equal to, or better than, the best heuristic solution in the initial population, because the best/fittest solution is always brought forward to the next generation.

The eight heuristics operate on batches of tasks, and each is presented with the same set of tasks. They are also all presented with estimated task execution times, estimated communication overheads, and execution rates of the processors in MFLOP. Details of how the task execution times and communication overheads are estimated can be found in [22]. We will now present each of these heuristics. The complexity of each of these heuristics is $\Theta \left(N^{2}\right)$, where N is the number of unmapped tasks and M is the number of processors. The complexity of the meta-heuristic proposed in this paper is also $\Theta \left(N^{2}\right)$.

The max–min (MX) heuristic begins with a set of unmapped tasks. The execution time of each task on each processor is added to an ETC matrix where ETC(i,j) denotes the execution time of task i on processor j. The ETC matrix is directly equivalent to the makespan. For each task, the processor which will compute it with the minimum amount of time is selected and added to a set. The task-processor mapping with the largest completion time in this set is selected. This task is then assigned to the processors queue, and removed from the set of unmapped tasks. This process is repeated until all tasks are mapped to a processor. The MX heuristic attempts to schedule the longest running tasks as early as possible, to processors which will process the tasks as fast as possible. Tasks with shorter execution times can then be mixed with the longer running tasks resulting in an overall move evenly balanced load across the processors and a better makespan.

The min–min (MM) scheduler [8] is similar to the MX heuristic, except that after the set of minimum completion times is found, the task with the overall minimum completion time is assigned to the corresponding processor. MM increases the probability that more tasks will get to execute on their first preference processor than with MX [18].

The max lightest loaded (LLX) heuristic scheduler considers the existing load on processors and the estimated MFLOP of the tasks. The set of unmapped tasks is sorted in descending order according to their estimated size. The task with the largest computational requirement (in MFLOP) is then assigned to the lightest loaded processor. This is repeated until all tasks have been mapped to processors. LLX does not consider the time a task will take to execute on a given processor. It instead aims to put large tasks on lightly loaded processors, and small tasks on heavily loaded processors. If the estimated processing time of tasks has a high error, this heuristic will still provide a reasonably distributed load compared to MX and MM.

The min lightest loaded (LLM) heuristic scheduler operates in the same way as LLX, except the computational requirements of the tasks are sorted in ascending order. It attempts to schedule the smallest tasks first to increase the throughput of tasks.

Each of the heuristics above, MX, MM, LLX, and LLM assume there is no network overhead for scheduling a task on a processor. Where the processing to communication (P-to-C) ratio is very high, the network overhead may be negligible, but when it is low, or when there is limited network resources, the communications overhead must be considered for scheduling a task on a processor.

A variant of each of the above heuristics, MXC, MMC, LLXC and LLMC estimates the communication cost of mapping tasks to processors. Communication costs are estimated using the k-NN algorithm as described in [22]. The makespan is updated to include communication costs, ETC(i,j)+C(i,j) where C(i,j) is the estimated communication overhead associated with executing task i on processor j. As each processed task is returned, a tuple of information (total communication time in seconds, task inputs, task identifier, processor identifier) is saved to the communications observation set. An estimated communication time in seconds is generated by passing in the processor identifier j, the task identifier i and the input parameters to the task. Apart from PN (overall meta-heuristic) MXC, MMC, LLXC and LLMC are the only new heuristics proposed in this paper. All other heuristics are proposed elsewhere.

Each heuristic is suited to different situations. MX performs well when there are more large tasks than small tasks, with MM performing better in the opposite situation [18]. LLX and LLM are ideal heuristics for the situation where the size of tasks to be processed is not known, or the estimated processing time has high error. The variations of all the heuristics, which estimate communication costs, allows for efficient schedules to be produced in systems with high communications costs, such as massively distributed systems.

### 2.4 Evolutionary phase

The evolutionary phase of the GA is governed by the cycle crossover method [20]. Two parent (A and B) strings are randomly selected from the population. Index $x_{1}$ is randomly chosen. $A_{x_{1}}$ and $B_{x_{1}}$ are marked as having been visited. The value contained in $B_{x_{1}}$ is noted. This value is then searched for in A and the index of this value is denoted as $x_{2}$. $A_{x_{2}}$ and $B_{x_{2}}$ are then marked as having been visited, and the value in $B_{x_{2}}$ is searched for in A. This continues until an index in A is visited twice. A cycle has now been found. All indices visited are then crossed over to produce 2 new child strings. This ensures that the child strings generated are valid, e.g. only 1 task may be scheduled to 1 processor at any time. Since both parents contain the exact same character, just in a different order, a cycle will always be found.

### 2.5 Mutation

Two types of mutation are employed by the GA, one randomly swaps elements of chromosomes in the population, and the other is a rebalancing heuristic. Random mutations are an essential part of a GA, perturbing the population to allow for new areas in the solution space to be searched. Every generation a percentage of elements in the population is randomly mutated. If the improvement in the makespan has not improved after 10 generations, the mutation rate is increased. Once the makespan begins to improve again the mutation rate is reduced. This reduces the probability of the GA getting stuck in a local minimum.

The other mutation operation utilizes a rebalancing heuristic to reduce the makespan. It achieves this by attempting to more evenly distribute the load on processors, by swapping tasks from heavily loaded processors on to lightly loaded processors. It has an average case complexity of Θ(M+N), where M is the number of processors, and N is the number of tasks. The solution generated by the heuristic will be discarded if it is worse than the starting solution, thus ensuring that the heuristic will only have a positive effect on the makespan.

### 2.6 Selection

The selection technique is based on the roulette wheel method [7,25,30]. The probability of a string going forward to the next generation is represented as a proportional sized slot on the roulette wheel, with a range from 0 to 1. Random numbers from 0 to 1 are then generated. The string which corresponds to the randomly selected slot is brought forward to the next generation. Since fitter strings have larger slots, they are more likely to be brought forward to the next generation. This process continues until a sufficient number of strings are selected.

### 2.7 Stopping conditions

When the stopping conditions are met, the evolution of the population will halt. This is to prevent the GA from running forever. Since this scheduler is intended for use in an on-line distributed system, it must produce schedules in a reasonable amount of time. Thus we use two stopping conditions: (1) there is an upper bound on the maximum number of generations, to guarantee evolution will halt and (2) if the makespan of the best solution has not changed after a set number of generations, then the GA will stop.

## 3 Experiments

For the experiments described in this section, we primarily used the 3 experimental setups in Table 1, run on a heterogeneous Java distributed system [11]. The first and simplest setup is a homogeneous set of processors, which we use as a base case for our experiments. This allows schedulers which favour a homogeneous set of processors to excel. The next setup is a set of processors with 2 homogeneous sets of processors. Both of theses setups used a 100 Mbps network. Finally, we used a set of processor with high heterogeneity and with a heterogeneous network which was spread over 3 different LANs and ranged from 10–100 Mbps. We had non-dedicated usage of these processors, and the actual available processing and network resources varied stochastically over time. All experiments were performed at off-peak times to minimize the effect of these variations. All the clients connected to a dedicated server running Linux (Fedora Core 4) on a 3 GHz P4 with 1 GB of RAM.

### 3.1 Other scheduling algorithms

The performance of the PN scheduler has been compared to the performance of a number of different schedulers. These schedulers are the most commonly used schedulers in distributed computing (see Table 2). The earliest first (EF) scheduler [17] is an immediate mode heuristic scheduler. It schedules tasks on the processor which will finish processing earliest. The lightest loaded (LL) scheduler is also an immediate mode scheduler, scheduling tasks on the most lightly loaded processors, without regard for the processing time of the task. MX is a batch scheduler which attempts to schedule the largest tasks first, and MM is the opposite, scheduling the smallest tasks first. We compare PN to three other evolutionary schedulers. A simulated annealing (SA) [14] based scheduler was created using the open source library Jannealer [9]. A tabu search (TA) based scheduler was created using OpenTS [21]. A GA scheduler (ZO) developed by Zomaya & Teh [30] is used for comparison purposes.

The scheduling algorithms are of varying complexity (see Table 7), from the least complex, round robin (RR), to the most complex evolutionary algorithms. These schedulers represent the most commonly used heuristics and the state-of-the-art evolutionary schedulers.

### 3.2 Heterogeneous distributed system

A general purpose programmable Java distributed system, which utilizes the free resources of a heterogeneous set of computers linked together by a network, has been developed [11]. The system has been successfully deployed on over 500 computers, which were distributed over a number of locations, and has been successfully used to process bioinformatics [13], biomedical engineering [23], and cryptography applications.

The distributed system consists of 3 Java archive files, a client, a server and a remote interface. A problem can be created for the system simply by extending 2 classes, called Algorithm and DataManager. The Algorithm class is run on the client and specifies the actual computation to be performed. The DataManager class is run on the server and specifies how the problem is broken up into tasks and how the processed results are recombined.

The distributed system provides a simple scheduling interface, which allows the administrator of the system to select a scheduling algorithm using the remote interface. To create a new scheduler, a programmer only needs to extend the SchedulerCommon API and implement a single method called generateSchedule. This method simply takes in a list of tasks and maps them to processors. The system defaults to the simplest scheduler, round robin.

Table 3 has a quick overview of the properties of each problem application. The set of problem used in this section is detailed in [22].

### 3.3 GA experiments

Parameters used within a GA, such as the number of generations, mutation rate and chromosome length, can effect the running time and quality of results generated by the GA. We will investigate the effect varying these can have on the scheduling algorithm.

**Fig. 2:**
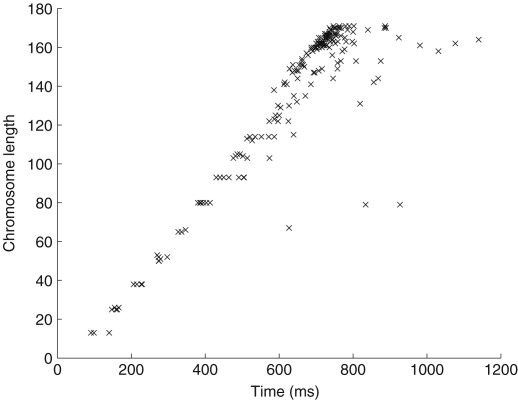
Execution time (ms) of PN scheduler with a fixed number of generations and a fixed mutation rate. The chromosome length corresponds to the number of tasks to be scheduled.

The execution time of the scheduler increases approximately linearly with an increase in the number of chromosomes. This can be seen in Fig. 2, where we varied the chromosome length and measured the execution time of the GA. We fixed the number of generations at 500 and ignored all other stopping conditions. The execution time of a given chromosome length varies, due to the stochastic nature of overheads in a real-world distributed system, but the majority of times fall into a tight linear range. The tasks used in the experiment are described in Table 3. The scheduler produces schedules for large numbers of tasks and processors quickly, for example, the GA scheduler can schedule a batch of 170 tasks in under 1 s.

**Fig. 3:**
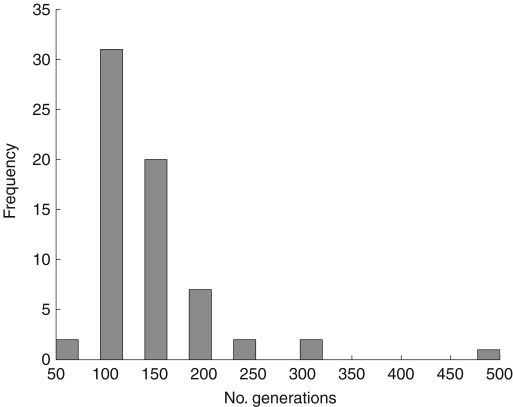
Number of generations run before stopping conditions terminate the evolution of the GA.

However, the scheduler uses a variable number of generations, depending on whether the stopping conditions are met. If there in no improvement after 50 generations, the algorithm stops. The figure of 50 was chosen as a large enough figure to allow for the random mutations to evolve a solution out of a local minima without impacting significantly on the running time of the algorithm. The histogram in Fig. 3 shows the number of generations performed before this stopping condition halts evolution. It forms a Poisson distribution, which indicates that the scheduler finds either a local minimum or the global minimum makespan within a relatively low number of generations.

**Fig. 4:**
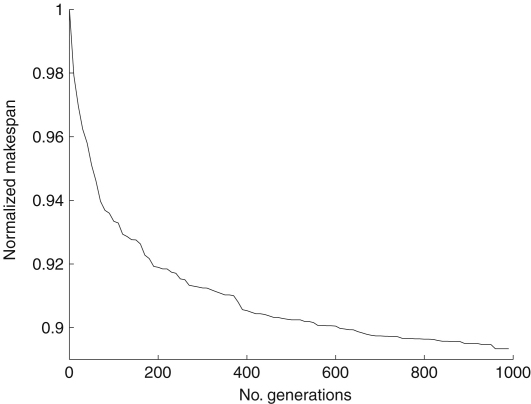
Average makespan achieved with varying numbers of generations in the GA scheduler.

When the quality of the solution produced is considered, we found that the greatest average reduction in makespan occurs within the first 200 generations. Fig. 4 shows this with a large reduction in makespan at the beginning, but the returns diminish quickly. Since the execution time of a generation is a constant factor, reducing the number of generations allows for a lower execution time of the scheduler. In a real-time system a client might be lying idle whilst waiting for a schedule to be produced, nullifying the effects of a more efficient schedule, thus a lower scheduler execution time is desirable.

We then looked at the effect the population size on the makespan achieved when scheduling on a real-world distributed system with 124 processors (see Table 4). Table 5 shows that when a larger population size is used, the effect on the overall makespan is negligible compared to using a small population size. This is due to the stopping condition which halts evolution if there is no improvement in makespan after 50 generations. The greater diversity in a large population allows for a minimum to be found in less generations, which offsets the longer execution time for a single generation. A smaller population requires more generations to achieve the same effect, however the execution time for each generation is less. The only difference between using a small and large population size is the spacial requirement. Thus to reduce the overall memory consumption of the algorithm we use a small population size (a micro-GA [3]).

### 3.4 Multiple heuristics performance

**Fig. 5:**
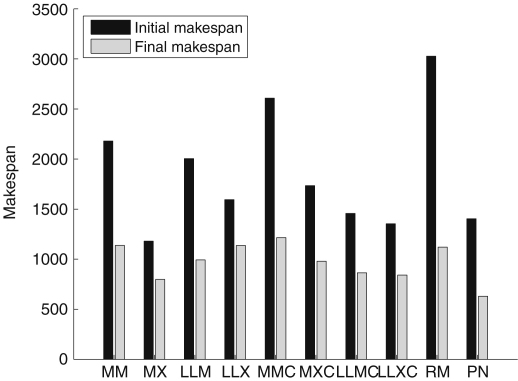
Performance of each heuristic when used on its own to initialize the GA.

We wish to show that using multiple heuristics to generate schedules for the initial population of the GA provides more efficient schedules than using each individual heuristic on its own, or using a purely random initial population. In Fig. 5 we use each heuristic individually to initialize the population of the GA. Each bar is an average 10 simulations, and we scheduled 600 tasks with normally distributed execution times on 30 heterogeneous processors.

The black bar shows the average initial makespan produced by the heuristic, and the gray bar corresponds to the average final makespan produced by the GA from that initial population. The population consists of only one heuristic and random variations of the schedule produced by the heuristic. A randomly chosen initial population (RM) presented for comparison purposes. The algorithm presented in this paper (PN) utilizes all of the heuristics to generate an initial population. The initial makespan for PN is an average of the best solutions generated by the heuristics. As can be seen in Fig. 5 using multiple heuristics provides, on average, a lower makespan.

**Fig. 6:**
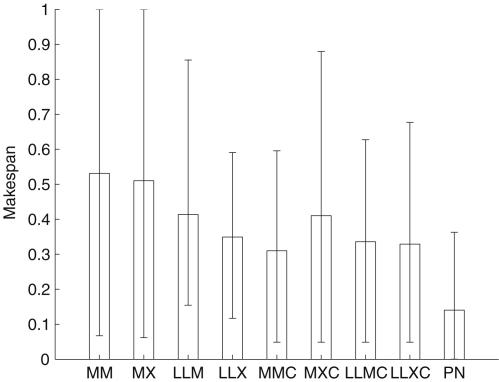
Performance of heuristics compared to our algorithm (PN) with real problems on a real heterogeneous distributed system, with normalized makespan.

In Fig. 6 we compared each heuristics initial solution to the final evolved solution (PN), with PN utilizing all of the heuristics. A set of 6 real-world problems (see [22] for details) were used for this experiment, processed by 25 non-dedicated heterogeneous processors (see Table 6). Fig. 6 shows the average initial solutions (normalized makespan) found by each heuristic after scheduling 60 different batches of tasks. The final evolved solution provides more efficient solutions on average than the solutions produced initially by the individual heuristics. The errorbars also show that the schedules produced by PN vary over a smaller range than the schedules produced by the other heuristics.

### 3.5 Performance evaluation

Each scheduler was presented with the same set of problems and the same set of processors (see Table 1). The makespan is measured as the time from when the first task is requested from thedistributed system, to the time when the final task is returned to the system. Table 7 shows that there is a huge difference in makespan (lower is better) with PN processing all tasks much faster than the next best scheduler when using a highly heterogeneous set of processors and networking resources. The variation in makespans can be accounted for by inefficient mappings of tasks to processors, such as slow processors being given computationally intensive tasks or processors with high communication overheads being given tasks with a low P-to-C ratio. The experiment was repeated with a set of resources that displayed low heterogeneity (see Table 1.B). With less heterogeneity the difference in makespan is only 13% between the best (PN) and the worst (SA). With high heterogeneity this difference was 132%, with PN generating the lowest makespan (Table 8).

When the experiment is repeated on a homogeneous set of processors the differences in makespan between the schedulers becomes negligible (see Table 9) with most schedulers utilizing the processing resources efficiently with up to 97% efficiency. PN, ZO and TA generate schedules which are within 1% of each other in this case and can adapt well to this homogeneous resource environment, which is to be expected. The simple heuristic schedulers generate solutions which have makespans which are 20%–38% longer than the evolutionary algorithms.

**Fig. 7:**
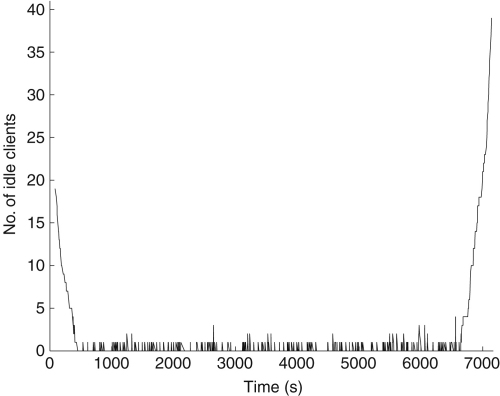
The number of idle clients in the system while the set of problems is being processed with the authors’ scheduling algorithm (PN).

Fig. 7 shows the number of idle clients while the set of problems is being processed using the PN scheduler in a highly heterogeneous resource environment. The initial assignment of tasks to processors does not happen instantaneously because the client machines only contact the server at set intervals (1 min in this case). Near the end when the steep slope shows that all of the clients stop processing tasks within a short interval. If this was a shallow slope it would indicate processing resources are idle and underutilized.

The overall scheduling framework used in this paper allows for a zero knowledge approach to be adopted. The target user audience for this scheduler consists of non-technical researchers, who want to create a distributed application and have it “just work” without having to worry about scheduling. As all properties of the system and the tasks to be processed are estimated on-line, there is no need for the user to provide a DAG, an ETC matrix in advance or to have previously executed the application.

The simple list scheduling heuristics (LL, EF, RR) take the next available task and schedule it. They do not allow for tasks to be scheduled out of sequence. The underlying algorithms are very simple, deterministic and easy to understand. The overheads are also quite low in complexity terms O(M), M is the number of processors, or in the case of RR O(1). The simple batch scheduling methods (MM, MX) can take a set of tasks and schedule them at once. This allows for the scheduler to look ahead to select the best task to assign to a processor. This additional capability only makes the heuristics slightly more complicated however it does increase the complexity to $O\left(N^{2}\right)$.

The evolutionary algorithm based schedulers (PN, ZO, TA, SA) can allocate batches of tasks to processors and utilize evolutionary techniques to find near optimal solutions. The algorithms can quickly traverse large solution spaces, which allows them to adapt to different resource and computational environments. The non-deterministic nature of the algorithms can limit the applicability of these methods, such as in time critical systems or medical systems. These techniques are also more complicated internally. They all require parameters to be set and inappropriately set parameters can have a detrimental effect on the quality of the solutions found. Whilst these methods can theoretically find near optimal solutions, this can require substantial amounts of time, for example, SA will find the optimal solution given infinite time. To ensure that these algorithms finish in a realistic amount of time, where processor idle time is minimized, stopping conditions must be imposed to cut off the algorithm and return the current best solution. These trade-offs when applied to the evolutionary heuristics result in differences in performance between the algorithms.

Tabu based algorithm (TA) works reasonably well in two of the three experiments. We intend to investigate using a hybrid of this algorithm for future scheduling research as its underlying similarity to genetic algorithms may well benefit from using a number of simple heuristics. The performance of the Simulated Annealing method is affected by the parameters required for the algorithm, such as the temperature and the cool down factor. For the experiments presented here, the SA parameters were set by using the simulated annealing algorithm itself, which is a standard way of setting the parameters. This is problematic however, because it has a tendency to work for a specific set of circumstances, and when faced with the unknowns of a real-world distributed system, it cannot adapt quickly enough.

Compared to other methods the GA based schedulers (PN, ZO) provide reasonably efficient solutions. PN significantly extends the ZO heuristic to work with a heterogeneous resource environment and unknown task execution time distributions. A downside to the PN algorithm is the complicated non-deterministic nature of the algorithm. A simpler algorithm is usually preferential over a complicated algorithm (Occams Razor). Also as it is non-deterministic, given the same set of inputs, it is unlikely that the same set of outputs will result. This limitation also affects the other evolutionary algorithms. Overall however, PN provides an algorithm which can provide efficient solutions in a wide variety of unknown task execution time distributions, and can adapt to heterogeneous resources. This best meets our objective to create a heterogeneous computing scheduler which can be used by non-technical users without the need for them to provide a priori knowledge of the resources or computational requirements.

## 4 Conclusion

A scheduler was developed for the task allocation problem in a dynamic heterogeneous distributed system. It is a multi-heuristic evolutionary algorithm, which utilizes a GA, to allocate tasks to processors in polynomial time. The use of eight heuristics to initialize the GA allowed for more efficient schedules to be created than would have been with a purely random initial population. If at any stage a processor becomes idle the scheduler returns the current best solution, which will always be at least as efficient as the best heuristic solution. The GA was implemented in Java and incorporated into a distributed system. A set of real-world problems from bioinformatics, biomedical engineering and cryptography was used to test the scheduler. Experiments were performed up to 150 heterogeneous processors, and show that the scheduler presented in this paper outperforms the most commonly used heterogeneous distributed computing scheduling heuristics. The more heterogeneous the resources of a system become, the harder it is to generate an efficient mapping of tasks to processors. We have presented an algorithm which achieves better efficiency than other schedulers as the resources become more heterogeneous.

For future work, the next logical step would be to distribute the scheduling algorithm to take full advantage of the available computational resources. Investigation is also needed into dynamically adapting task execution time distribution estimation techniques to identify the characteristics of applications at runtime which may yield less erroneous computational requirements estimations. Task dependencies will also need to be considered, allowing for this work to be applied to a larger set of problems. We also intend to investigate the use of different heuristics, dynamically changing the set of heuristics at runtime based on observed performance.

The distributed system software is freely available under an open source GNU GPL license from the system homepage located at http://www.cs.nuim.ie/distributed.

## Figures and Tables

**Table 1:** Client resources of different experimental setups.

	Heterogeneity	No. proc	MFLOP/s	RAM (MB)	O/S	Processor type
A	High	91	28–31	256	Linux	P3 600 MHz
		50	190–229	512	Linux	P4 2.4 GHz
		4	15	192	Linux	P2 266 MHz
		1	154	1024	Windows	Centrino 1.4 GHz
		1	25	512	Linux	P3 500 MHz
		1	37	256	Linux	P3 1 GHz
		1	72	256	Linux	P4 1.7 GHz
		1	91	1024	FreeBSD	AMD 2400+XP
B	Low	45	28–31	256	Linux	P3 600 MHz
		45	180–200	1024	Linux	P4 D820
C	Homogeneous	45	180–200	1024	Linux	P4 D820

**Table 2:** Taxonomy of schedulers.

Type	Key	Name	Reference
Immediate	RR	Round Robin	
	EF	Earliest first	[17]
	LL	Lightest loaded	
Batch	MM	Min–min	[8]
	MX	Max–min	[8]
Evolutionary	SA	Simulated annealing	[14]
	ZO	Zomaya–Teh	[30]
	TA	Tabu search	[5]
	PN	This paper	Algorithm 1

**Table 3:** Comparison of problem properties.

Problem	Avg communication time (s)	Avg processing time (s)	P-to-C	No. tasks	Reference
SLTT	12.4	519.81	41.92	143	[23]
DSEARCH	14.0	731.99	52.05	612	[12]
MD5	14.4	235.52	16.36	800	[19]
SHA1	64.5	543.02	8.42	900	[26]
Elgamal	29.2	419.96	14.34	406	[4]
TSP	9.5	353.72	37.04	121	

**Table 4:** Client resources used in the distributed system for the experiment shown in Table 5. The operating system on all clients was Linux.

No. processors	MFLOP/s	RAM (MB)	Network link (Mb/s)	Processor type
47	180–200	1024	100	P4 D820
45	190–229	512	100	P4 2.4 GHz
32	28–31	256	10	P3 600 MHz

**Table 5:** Varying population size of the scheduling algorithm where the GA terminates if there is no improvement in makespan after 50 generations.

Population size	Makespan (s)	Scheduling time (s)	Mean scheduling time (s)	% Efficiency	% Communication costs
10	4653	24.0	0.31	80.4	0.407
20	4720	23.1	0.30	78.1	0.405
30	4701	26.3	0.30	86.4	0.444
40	4672	22.6	0.28	86.9	0.436
50	4649	29.7	0.33	83.3	0.436
60	4846	32.1	0.34	84.3	0.437
100	4686	25.7	0.31	80.9	0.463
1 000	4855	32.3	0.34	86.3	0.541
5 000	4720	24.8	0.31	83.1	0.418
10 000	4711	21.5	0.28	82.7	0.415

**Table 6:** Client resources used in the distributed system for the experiment shown in Fig. 6.

No. proc	MFLOP/s	RAM available (MB)	O/S	Network link (Mb/s)
9	214	257–296	Windows	10
7	244	100	Windows	100
3	255	261–265	Windows	10
2	223	257–267	Windows	10
2	255	100	Windows	100
1	32	100	Windows	10
1	221	64	Linux	100

**Table 7:** Comparison of schedulers with a set of highly heterogeneous processors and a heterogeneous set of networking resources.

Scheduler	Makespan (s)	Scheduling time (s)	Mean scheduling time (s)	% Efficiency	% Communications	% Inefficiency
This paper (PN)	9 144	138.2	2.239	53.01	2.84	0
Zomaya–Teh (ZO)	14 278	276.1	1.904	33.94	2.24	56
Tabu (TA)	16 378	322.8	4.818	33.66	2.34	79
Sim. annealing (SA)	21 260	4605.4	47.478	30.07	5.95	132
Max–min (MX)	15 486	0.5	0.006	34.94	1.84	69
Min–min (MM)	18 321	0.4	0.005	32.21	2.30	100
Lightest loaded (LL)	19 645	0.1	0.001	25.05	1.82	114
Earliest first (EF)	14 492	0.4	0.004	46.88	10.96	58
Round Robin (RR)	20 314	0.1	0.001	31.90	9.10	122

**Table 8:** Comparison of schedulers with a set of 2 types of homogeneous processors and a heterogeneous set of networking resources.

Scheduler	Makespan (s)	Scheduling time (s)	Mean scheduling time (s)	% Efficiency	% Communications	% Inefficiency
This paper (PN)	8437	60.2	0.506	92.5	1.1	0
Zomaya–Teh (ZO)	8593	39.4	0.210	90.6	0.9	2
Tabu (TA)	8767	39.5	0.376	88.4	1.4	4
Sim. annealing (SA)	9564	3938.3	17.27	84.1	7.4	13
Max–min (MX)	9065	0.091	0.0008	87.3	1.1	7
Min–min (MM)	8860	0.166	0.0013	87.0	1.3	5
Lightest loaded (LL)	9053	0.021	0.0002	87.0	0.9	7
Earliest first (EF)	8602	0.089	0.0007	90.9	1.1	2
Round Robin (RR)	8812	0.096	0.0006	88.4	0.9	4

**Table 9:** Comparison of schedulers with a homogeneous set of processors.

Scheduler	Makespan (s)	Scheduling time(s)	Mean scheduling time (s)	% Efficiency	% Communications	% Inefficiency
This paper (PN)	10 408	50.4	0.49	96.9	1.3	1
Zomaya–Teh (ZO)	9 969	21.7	0.17	97.6	1.2	0
Tabu (TA)	10 126	22.3	0.23	97.5	1.3	1
Sim. annealing (SA)	10 351	1530.5	12.24	95.2	3.2	1
Max–min (MX)	12 034	0.05	0.01	81.8	1.1	20
Min–min (MM)	13 788	0.04	0.01	69.9	0.8	38
Lightest loaded (LL)	13 841	0.01	0.01	69.9	0.8	38
Earliest first (EF)	13 836	0.03	0.01	69.7	0.8	38
